# The Understanding of Peak Oxygen Uptake in Children Aged 8–16

**DOI:** 10.3389/fped.2020.599571

**Published:** 2021-01-14

**Authors:** Simpson W. L. Wong, Clare C. W. Yu, Albert M. Li

**Affiliations:** ^1^Department of Education Studies, Hong Kong Baptist University, Hong Kong, China; ^2^Department of Rehabilitation Sciences, The Hong Kong Polytechnic University, Hong Kong, China; ^3^Department of Paediatrics, The Chinese University of Hong Kong, Hong Kong, Hong Kong, China

**Keywords:** cognitive development, conservation, perfactual thinking, cardiovascular fitness, peak oxygen uptake, physical literacy

## Abstract

**Objective:** To examine the understanding of the concept peak oxygen uptake (peak VO_2_) among children and adolescents at different ages from a developmental perspective.

**Methods:** A total of 549 children and adolescents aged 8 to 16 were recruited and instructed to fill in a 20-item *Peak VO*_2_ *Understanding Inventory* developed with reference to the research literature on peak VO_2_. We presented the participants with twenty scenarios and asked them to indicate whether peak VO_2_ would “remain unchanged,” “increase,” or “decrease,” or that there was “insufficient information for a definite answer.” The cross-sectional data was analyzed by employing a series of ANOVA analyses and chi-square association tests. Additional statistical analyses were performed to examine the error patterns and if there were gender differences.

**Results:** Except for the 8-year-old group, the overall accuracy rate did not improve with age. Age-related differences in the choice of answers (“increase,” “decrease,” “unchanged,” and “uncertain”) for determining the resulting peak VO_2_ after a change of antecedent were observed. Error analysis by item showed that prefactual thinking that is important to understand the concept was emerging rather than fully developed in our child and adolescent samples.

**Conclusion:** The mastery of peak VO_2_ is not subject to age-related maturation but might demand the acquisition of specific logical reasoning skill such as perfactual thinking. Early introduction of peak VO_2_ and related concepts is advocated and should be emphasized on the reasoning rather than providing model answers in physical literacy education.

## Introduction

Cardiopulmonary fitness is one of the most essential dimensions of health-related fitness, partly because of its close association with cardiovascular risk factors such as abdominal adiposity in children and adolescents (1, 2). According to several international standards, regular daily moderate-to-vigorous physical activity (MVPA) is recommended to ensure cardiopulmonary fitness. In spite of the well-known recommendations and the benefits of MVPA, many people still lead a sedentary lifestyle. One attempt to understand people's attitude toward physical activity is through a comprehensive examination of their physical literacy (3). To date, physical literacy is regarded as a multi-dimensional construct, encapsulating a series of parameters that explain an individual's participation in physical activities for life (4). Given the multi-faceted nature of physical literacy, a collection of assessment tools rather than a single measure are used for the evaluation of physical literacy. Among these tools, self-reported inventories are the most commonly used method. It has been shown in an empirical study that there was a significant correlation between the perceived and actual physical literacy (5). However, even if a person confidently reports that he/she is aware of the importance of the health concept (e.g., peak VO_2_) and believes they have enough knowledge about the concept, it is uncertain if this individual's belief is true. A direct measure of their health literacy can clarify this self-perception bias.

Among various indicators of cardiopulmonary fitness, the highest oxygen uptake elicited in a maximal exercise test to exhaustion is widely considered as the benchmark for such fitness assessment (6). This parameter is commonly referred to either as maximal oxygen uptake (VO_2_ max) or as peak oxygen uptake (peak VO_2_); herein, the term “peak VO_2_” is used. Despite the significance of peak VO_2_, little is known about whether this concept can be fully understood by children and adolescents. To the best of our knowledge, children and adolescents' understanding of peak VO_2_ has not been tested in any research study. Without this knowledge, it is not easy to plan the physical literacy curriculum designed for students at different years of age. The current study aims to fill these research and pedagogical gaps in health literacy.

By definition, peak VO_2_ is an abstract concept and thus cannot be observed with the naked eye. However, it is measurable through a maximal graded exercise test. In children, who are not health professionals and have only a limited basic knowledge of the human body, understanding the concept of peak VO_2_ requires several mental skills. The definition of peak VO_2_ includes a linguistic marker “superlative” (“highest” and “maximal”) which may be difficult to comprehend for young children. Some notion of “oxygen,” as the element that we breathe in to sustain our lives, is requisite. The ability to imagine the flow of oxygen through the cardiopulmonary system is also required to understand peak VO_2_. As a health concept, people's knowledge of the link between peak VO_2_ and its determinants is essential. Therefore, we examined peak VO_2_ by relating this concept to “if-then” conditional propositions structuring a causal relationship; that is, “if (cause, i.e., determinant), then (effect, i.e., the resulting peak VO_2_).” Using “if-then” conditional propositions, we generated a series of hypothetical situations for testing children and adolescents' understanding of peak VO_2_.

Another main reason for formulating questions as “if-then” conditional propositions was that such propositions guide goal-directed behaviors. The logic behind processing such propositions is *prefactual thinking*, (i.e., thinking how things might be different in the future). Thoughts assume the form of “if-then” conditional propositions in which “if” specifies an alternative action or condition (e.g., “if I do more exercise”) and “then” specifies the imagined outcome or goal (e.g., “then my peak VO_2_ will increase”). Accordingly, such thinking enables consideration of different courses of action that may have led, or may still lead, to a more desirable outcome, preparing one to implement such actions through a range of future-oriented cognitions if a comparable situation arises in the future. Furthermore, prefactual thoughts are essential because they focus on controllable elements (e.g., concentration level or specific strategies to improve skills). By imagining how things might be different in the future, people become more aware that the constraints governing their previous attempts (task demands, available resources, and their own skills) will also be present in the future.

The hypothetical scenarios tested in this study were created with reference to research on peak VO_2_. In those empirical studies, a linear increase in boys' peak VO_2_ was observed from 8 to 18 years of age. In girls, the trend was similar but less consistent than boys' values, which tended to plateau in the midteens (7). Peak VO_2_ was higher in boys than in girls, with the difference between them becoming significant after 12 years of age and gradually widening with increasing age (8, 9). In adulthood, peak VO_2_ started to decline with advancing age in both men and women (10–12). Although people who are overweight or obese did not have an impaired peak VO_2_ relative to fat-free mass, individuals with obesity had a reduced peak VO_2_ relative to body weight (13). Additionally, hyperthermia caused by exercising in a hot environment impaired peak VO_2_ (14–16). Moreover, peak VO_2_ decreased with the partial pressure of inspired oxygen at high altitudes (17). Although prolonged periods of aerobic training improved peak VO_2_ considerably, it decreased within weeks after training cessation (18). The reviewed literature provided the basis for our development of the inventory used in this study.

As shown in previous research, concept learning is limited by age-graded cognitive development. According to Piaget's model of cognitive development, one developmental milestone is the ability to understand hypothetical situations (19). Given the volume nature of peak VO_2_, the Piagetian idea of *conservation* is useful to conceptualize the level of understanding of peak VO_2_. Conservation, defined as the ability to mentally preserve the attribute of an object/an observation despite the change of appearance, is critical for performing mental computation on a wide range of dimensions such as volume, length, area, etc. Jean Piaget developed a cognitive development model with four successive stages: sensorimotor stage (roughly 0–2 years), pre-operational stage (roughly 2–7 years), concrete operational stage (roughly 7–11 years) and the formal operational stage (11 years and beyond). Along these stages, children's thinking changes from concrete to abstract. They develop the ability to replace overt actions by mental representations, with egocentrism and centration diminishes. Children develop more eye for detail, their information processing capacities increase, and their problem solving becomes more and more advanced. Based on the previous studies of conservation and the model of cognitive development, we hypothesize that the understanding of peak VO_2_ differs in children of different ages. Hence, a cognitive development perspective was adopted to examine the understanding of peak VO_2_ in school children aged 8–16 years. The results will provide insight into age-related differences in the interpretation of peak VO_2_.

Gender differences were noted in previous study. In general, girls were found to be less active and skillful in movement during physical exercise (PE) lesson. Owing to lower levels of perceived motor competence, girls did not enjoy as much as boys did in PE lessons (20). Such gender differences have prompted curricular designers to include arts-based teaching in PE lessons, such as dance (21). Apart from the environmental change, it is imperative to identify the cognition in girls that may hinder or facilitate their participation in a board range of physical activities. In this study, we examined if girls' and boys' understanding of peak VO_2_ was different.

Extended from previous studies that showed the link between peak VO_2_, its determinants and the resulting health conditions, this study examines the understanding of peak VO_2_ among children and adolescents. The major research question that motivates the current study is: “What is the understanding of peak VO_2_ in children aged 8–16 years old?” Using a cross-sectional design, we tested nine groups of participants ranging from 8 to 16 years old. This research design allows the examination of age-related differences, which is critical for identifying the optimal time and level of difficulty for introducing the concept of peak VO_2_. A more specific research question is: Does age affect the extent to which a student understands the concept of peak VO_2_? According to the stage theory of cognitive development (19), children over 6 (in the “pre-operational” stage) should be able to understand concepts involving conservation ability. These children are expected to be able to decide when peak VO_2_ remained unchanged, even something observable has been altered. Elder children, about 7–12 years old, will be able to apply inductive reasoning to solve an abstract question (in the “concrete operational” stage). Twelve-year-old or above have more advanced logical thinking. They can apply deductive reasoning to solve problems (in the “formal operational” stage). We answered this question by analyzing the accuracy and the choice of answers. Further to accuracy, we planned to infer the student participants' way of thinking when tackling the questions about peak VO_2_. We conducted error analyses to examine how the antecedent and the resulting peak VO_2_ were perceived by students of different ages. The next research question is: “Is there gender difference in the understanding of the concept of peak VO_2_?” We will compare the accuracy and the choice of answers between the male and female groups, with the consideration of age.

## Materials and Methods

Participants in this study were recruited from 12 primary and 19 secondary schools in Hong Kong through stratified sampling. These schools are mainstream schools that admitted students with normal IQ scores. Ethics approval was obtained from ethics committee of the Joint Chinese University of Hong Kong - New Territory East Cluster Clinical Research Ethics Committee (CRE Ref. No. 2013.574). Before data collection, informed written consents were obtained from the student participants and their parents. On the day of testing, the experimenters informed the children the nature of the testing and their rights, such as voluntary participation. Participants were tested individually in a quiet meeting room by trained Psychology undergraduate students under the supervision of the authors of this paper. The whole testing session was normally last for 15 minutes.

The *Peak VO*_2_ *Understanding Inventory* was drafted, revised and confirmed by the authors. First, we brainstormed a range of possible and impossible conditions in which peak VO_2_ may increase, decrease or remain unchanged. The inventory items shared a standard format eliciting *prefactual thinking* [if (under a circumstance), then (resulting peak VO_2_)]. After a careful selection, a total of 20 items were selected to be included in the inventory (Table 1). Each item presented the participants with a scenario, and they were asked to indicate whether peak VO_2_ would “remain unchanged,” “increase,” or “decrease,” or that there was “insufficient information for a definite answer.” The items described three main types of change: (a) environmental change (6 items), referring to a change in the external environment; (b) behavioral change (7 items), referring to a change in a person's actions; and (c) bodily change (6 items), referring to a change in internal physical condition. The remaining item was about general knowledge on peak VO_2_. Before the participants answering the twenty questions, they were introduced with the concept peak VO_2_ and its formal definition. Each correctly answered item was scored one point. The maximum score for this inventory was 20 points. The reliability of the scale, in terms of internal consistency and assessed by the Cronbach's alpha test, is moderate [α = 0.60 (22)].

**Table 1:** Items of the Peak VO_2_ Understanding Inventory and corresponding answers.

**Types of factors**	**Item no**.	**Items**	**Answers: Peak VO_**2**_ will become**
Environmental	1	When the oxygen level in the air increases,	Unchanged
	2	When in the mountains where the air is thin,	Decrease
	3	When inside an oxygenated compartment,	Unchanged
	4	When the surrounding air is oxygen-depleted,	Decrease
	5	When doing exercise in an extremely high temperature,	Decrease
	6	When the peak VO_2_ analyzer malfunctions,	Uncertain
Behavioral	7	When a person does high-intensity aerobic exercise on a regular basis,	Increase
	8	After short, vigorous exertion such as chasing a bus,	Unchanged
	9	When a person plays an exciting game in an amusement park,	Unchanged
	10	When a person wants to intentionally increase peak VO_2_,	Unchanged
	11	When a person falls into deep sleep at night,	Unchanged
	12	When a person does exercise regularly but then stops for a few months,	Decrease
	13	After taking a pro-cardiovascular health supplement,	Uncertain
Bodily	14	When a person gains weight and becomes obese,	Decrease
	15	When a person loses weight,	Uncertain
	16	When a person's cardiovascular function improves,	Increase
	17	When a person's physical condition becomes worse because of illness,	Decrease
	18	When a person grows from adolescence to adulthood,	Increase
	19	When a person grows from adulthood to old age,	Decrease
General knowledge	20	In general, the peak VO_2_ in boys is (higher/lower) than in girls.	Higher

### Statistical Analysis

Data are presented as means, standard deviations or percentage. The one-way analysis of variance (ANOVA) with age group as the between-subject factor, followed by *post hoc* Tukey's HSD tests, was performed to compare the performance of the inventory across different age groups. Chi-squared tests were conducted to examine the association between age (8–16 years) and response (1 = increase, 2 = unchanged, 3 = decrease, 4 = uncertain). Upon a significant Pearson chi-squared value (*p* < 0.05) was detected, *post hoc* analyses were performed to identify which cell had significantly more or fewer count as expected. A standardized residual larger than 1.96 indicated a significant difference at 95% confidence interval. A positive value exceeding 1.96 indicated that the actual count was more than the expected count; whereas, a negative value < −1.96 indicated that the count was fewer than the expected count.

## Results

The final sample comprised 549 children and adolescents. The characteristics of each group are presented in Table 2. The descriptive statistics of the peak VO_2_ inventory among the nine age groups are presented in Table 3. A two-way analysis of variance (ANOVA) was computed to test the effect of age and gender using General Linear Model analysis in SPSS. There was a significant main effect of age on the peak VO_2_ inventory total scores, *F*_(8,531)_ = 3.91, *p* < 0.05, partial η^2^ = 79. There was a nonsignificant main effect of gender on the peak VO_2_ inventory total scores, *F*_(1,531)_ = 0.09, *p* = 0.76. The age x gender interaction effect was not significant, *F*_(8,531)_ = 0.71, *p* = 0.68. A *post hoc* Tukey's HSD test further revealed that the youngest age group (i.e., 8-year-old group) performed significantly worse than did the 11-year-old (*p* < 0.05), 14-year-old (*p* < 0.05), and 15-year-old (*p* < 0.01) groups.

**Table 2:** Number of children in each age group (ranging from 8 to 16 years old).

**Age group**	**Age (month) (mean, S.D.)**	**N (count)**	***N* (%)**	**Gender ratio (male: female)**
8	103.69 (3.46)	59	10.7	1:1.1
9	112.95 (3.56)	70	12.8	1:1
10	125.69 (3.61)	63	11.5	1:0.9
11	137.81 (3.25)	60	10.9	1: 1.85
12	150.93 (2.96)	46	8.4	1:1
13	161.67 (3.80)	73	13.3	1:0.97
14	173.37 (3.57)	51	9.3	1:1.12
15	185.87 (3.21)	70	12.8	1:0.94
16	197.01 (3.88)	57	10.4	1:0.9
Total	149.43 (31.07)	549	100.0	1:1.05

**Table 3:** Descriptive statistics of total scores for the Peak VO_2_ Understanding Inventory.

**Age group**	**Mean (S.D.)** **in both males and females**	**Mean (S.D.)** **in males**	**Mean (S.D.)** **in females**	***t*-statistics *t* (df); *p*-value**
8	7.41 (2.62)	7.68 (2.81)	7.16 (2.46)	*t* (57) = 0.75; *p* = 0.45
9	8.00 (2.84)	8.43 (2.80)	7.57 (2.87)	*t* (68) = 1.26; *p* = 0.21
10	8.32 (2.00)	8.42 (1.93)	8.20 (2.10)	*t* (61) = 0.44; *p* = 0.66
11	9.10 (2.29)	8.71 (2.79)	9.31 (1.98)	*t* (58) = −0.95; *p* = 0.34
12	8.11 (2.64)	7.57 (2.35)	8.65 (2.85)	*t* (44) = −1.41; *p* = 0.16
13	8.59 (2.43)	8.59 (2.91)	8.58 (1.85)	*t* (71) = 0.02; *p* = 0.98
14	8.96 (2.53)	9.08 (3.02)	8.85 (2.07)	*t* (49) = 0.32; *p* = 0.74
15	9.07 (2.64)	9.25 (2.59)	8.88 (2.72)	*t* (68) = 0.57; *p* = 0.56
16	8.74 (2.87)	8.73 (2.65)	8.74 (3.15)	*t* (55) = −0.01; *p* = 0.99
Total	8.48 (2.59)	8.52 (2.67)	8.44 (2.51)	*t* (547) = 0.36; *p* = 0.71

The percentage of correct count for each item across the nine age groups is presented in Table 4. Results of chi-square tests on items of environmental change, behavioral change, and bodily change are presented as follows:

**Table 4:** Percentage correct of individual item in the Peak VO_2_ Understanding Inventory among children between 8–16 years old.

**Question/age**	**8 yo**	**9 yo**	**10 yo**	**11 yo**	**12 yo**	**13 yo**	**14 yo**	**15 yo**	**16 yo**	**Total**
Q1	15.3	22.9	28.6	21.7	21.7	24.7	41.2	34.3	49.1	28.6
Q2	44.1	52.9	55.6	68.3	58.7	60.3	60.8	57.1	38.6	55.2
Q3	13.6	27.1	33.3	33.3	32.6	38.4	35.3	40.0	45.6	33.3
Q4	66.1	68.6	74.6	73.3	71.7	61.6	54.9	57.1	49.1	64.1
Q5	16.9	18.6	14.3	15.0	13.0	15.1	21.6	11.4	8.8	14.9
Q6	15.3	5.7	11.1	13.3	13.0	11.0	3.9	7.1	7.0	9.7
Q7	33.9	34.3	28.6	30.0	23.9	17.8	19.6	30.0	28.1	27.5
Q8	27.1	42.9	33.3	35.0	37.0	42.5	21.6	38.6	42.1	36.1
Q9	16.9	27.1	25.4	16.7	15.2	16.4	19.6	20.0	24.6	20.4
Q10	27.1	22.9	30.2	31.7	34.8	54.8	52.9	40.0	38.6	37.0
Q11	30.5	31.4	34.9	41.7	19.6	35.6	25.5	35.7	40.4	33.3
Q12	49.2	57.1	60.3	56.7	52.2	60.3	66.7	58.6	63.2	58.3
Q13	37.3	42.9	30.2	51.7	39.1	41.1	37.3	38.6	40.4	39.9
Q14	32.2	10.0	30.2	25.0	30.4	23.3	31.4	18.6	19.3	23.9
Q15	67.8	77.1	81.0	85.0	69.6	80.8	84.3	80.0	86.0	79.2
Q16	54.2	52.9	58.7	70.0	60.9	67.1	64.7	68.6	61.4	62.1
Q17	45.8	57.1	54.0	63.3	63.0	54.8	64.7	71.4	61.4	59.4
Q18	54.2	57.1	61.9	78.3	67.4	68.5	84.3	80.0	68.4	68.7
Q19	32.2	21.4	20.6	35.0	26.1	31.5	35.3	48.6	26.3	31.0
Q20	32.2	21.4	20.6	35.0	26.1	31.5	35.3	48.6	26.3	31.0

### Environmental Factors

Concerning the item “What will be the peak VO_2_, when the oxygen level in the air increases” [Q1, unchanged; $\chi_{\left(24\right)}^{2}$ = 43.49, *p* < 0.01 (0.009)], *post hoc* comparison indicated that significantly more participants in the 8-year-old group chose the option “uncertain” (standardized residual = 2.7), whereas the 16-year-old group chose the correct answer “unchanged” significantly more often (standardized residual = 2.9).

Regarding the item “What will be the peak VO_2_, when in the mountains where the air is thin” [Q2, decrease; $\chi_{\left(24\right)}^{2}$ = 39.93, *p* < 0.05 (0.02)], *post hoc* comparison indicated that significantly more participants in the 9-year-old group chose the option “uncertain” (standardized residual = 2.8), whereas significantly more participants in the 16-year-old group chose the correct answer “decrease” (standardized residual = 2.5).

For the item “What will be the peak VO_2_, when inside an oxygenated compartment” [Q3, unchanged; $\chi_{\left(24\right)}^{2}$ = 31.61, *p* = 0.13], participants in all age groups had similar response frequencies.

Concerning the item “What will be the peak VO_2_, when the surrounding air is oxygen-depleted” [Q4, decrease; $\chi_{\left(24\right)}^{2}$ = 55.54, *p* < 0.001 (0.000)], significantly more participants in the 16-year-old group gave the correct answer “decrease” (standardized residual = 3.9).

Regarding the item “What will be the peak VO_2_, when doing exercise in an extremely high temperature” (Q5, decrease), no significant difference in the frequency of answers across the eight age groups was observed [$\chi_{\left(24\right)}^{2}$ = 29.45, *p* = 0.20].

For the item “What will be the peak VO_2_, when the peak VO_2_ analyzer malfunctions” [Q6, uncertain; $\chi_{\left(24\right)}^{2}$ = 57.27, *p* < 0.001], significantly more 9- and 10-year-old participants chose the option “increase” (standardized residual = 2.5 and 2.3, respectively), and significantly more 16-year-old participants chose “unchanged” (standardized residual = 2.0). Compared with other age groups, the 15-year-old group chose the option “uncertain” significantly more often (standardized residual = 2.5).

### Behavioral Factors

The participants were then asked, “What will be the peak VO_2_, when a person does high-intensity aerobic exercise on a regular basis?” (Q7, increase). Significantly more 9-year-old participants chose the option “uncertain” [$\chi_{\left(24\right)}^{2}$ = 51.22, *p* < 0.01, standardized residual = 4.7].

Next, the participants were asked, “What will be the peak VO_2_, after short, vigorous exertion such as chasing a bus?” (Q8, unchanged). No significant difference across all age groups was observed [$\chi_{\left(24\right)}^{2}$ = 36.01, *p* = 0.05]. Similar results were obtained for other scenarios in this category: “What will be the peak VO_2_, when a person plays an exciting game in an amusement park” [Q9, unchanged; $\chi_{\left(24\right)}^{2}$ = 27.70, *p* = 0.59]; “What will be the peak VO_2_, when a person wants to intentionally increase peak VO_2_” [Q10, unchanged; $\chi_{\left(24\right)}^{2}$ = 28.41, *p* = 0.24]; “What will be the peak VO_2_, when a person falls into deep sleep at night” [Q11, unchanged; $\chi_{\left(24\right)}^{2}$ = 35.14, *p* = 0.06]; and “What will be the peak VO_2_, when a person does exercise regularly but then stops for a few months” [Q12, decrease; $\chi_{\left(24\right)}^{2}$ = 28.24, *p* = 0.25].

However, for the item “What will be the peak VO_2_, after taking a pro-cardiovascular health supplement” (Q13, uncertain) a significant group difference was found [$\chi_{\left(24\right)}^{2}$ = 57.99, *p* < 0.001]. *Post hoc* comparison revealed that significantly more 16-year-olds chose “unchanged” (standardized residual = 2.1), significantly more 8-year-olds chose “decrease” (standardized residual = 2.6), and significantly more 13-year-olds chose “uncertain” (standardized residual = 2.5).

### Bodily Factors

For the item “What will be the peak VO_2_, when a person gains weight and becomes obese” (Q14, decrease), the pattern of responses across all groups was not significant [$\chi_{\left(24\right)}^{2}$ = 29.82, *p* = 0.19].

For the item “What will be the peak VO_2_, when a person loses weight” (Q15, uncertain), a significant group difference was observed [$\chi_{\left(24\right)}^{2}$ = 46.47, *p* < 0.01]. *Post hoc* analysis indicated that significantly more participants chose “increase” in the 9-year-old group (standardized residual = 2.4) and “unchanged” in the 15-year-old group (standardized residual = 2.6).

Regarding the item “What will be the peak VO_2_, when a person's cardiovascular function improves” (Q16, increase), no significant difference was observed [$\chi_{\left(24\right)}^{2}$ = 28.27, *p* = 0.24].

A significant overall group difference was found for the item “What will be the peak VO_2_, when a person's physical condition deteriorates because of illness” [Q17, decrease; $\chi_{\left(24\right)}^{2}$ = 36.40, *p* < 0.05]. However, *post hoc* analysis revealed no specific group difference.

No significant group difference was observed for the remaining two items: “What will be the peak VO_2_, when a person grows from adolescence to adulthood” [Q18, increase; $\chi_{\left(24\right)}^{2}$ = 29.14, *p* = 0.21] and “What will be the peak VO_2_, when a person grows from adulthood to old age” [Q19, decrease; $\chi_{\left(24\right)}^{2}$ = 34.26, *p* = 0.08].

Regarding the item about gender difference, “the peak VO_2_ in boys is (higher/lower) than in girls” [Q20, higher; $\chi_{\left(24\right)}^{2}$ = 26.57, *p* = 0.32], no group difference was found.

### Error Analysis by Gender Group

To identify if there was recurring patterns of errors specific to gender, the same non-parametric “age (8–16 years old) x answer options (increase, decrease, unchanged, uncertain)” chi-square test was conducted on the two gender groups independently.

In female students, seven significant differences were observed (chi-square < 0.05 or below). When asked about the resulting peak VO_2_, the ten-year-old females were more inclined to think that the peak VO_2_ remained unchanged (standardized residual = 2.0) while the 13-year-old females were more inclined to think that the peak VO_2_ was uncertain. The results further showed that, the 8-year-old and 10-year-old females were inclined to choose the answer option “uncertain” for the three questions they answered incorrectly (standardized residuals = 2.0–3.5).

In male students, when they were asked about the resulting peak VO_2_ in an extremely hot environment, the 12- and 14-year old students were more inclined to respond “uncertain” (standardized residuals = 2.1 and 2.3, respectively) while the 9-year-old students were more likely to suggest an increase (standardized residuals = 2.4). The 8-year-old males were more inclined to think that peak VO_2_ would increase after taking some pro-cardiovascular health supplement (standardized residuals = 2.1). The 16-year-old males are inclined to suggest that peak VO_2_ remains unchanged when a person loses weight (standardized residuals = 2.8). When asked about the resulting peak VO_2_ after a person has just completed some vigorous exercise, 9-, 12-, and 15-year-old males were inclined to suggest “increase,” “decrease” and “uncertain,” respectively (standardized residuals = 2.2, 2.1, 2.0, respectively). The 9-year-old males responded relatively more “uncertain” when asked if a person stays on a mountain for some time (standardized residual = 4.2). Interestingly, when asked what would happen if a person staying inside an oxygenated compartment, the 9-year-old males reported relatively more “decrease” (standardized residual = 2.0) and somewhat less “increase” (standardized residual = −2.1), implying polarized opinion in this age group.

## Discussion

The primary aim of this cross-sectional study was to examine the understanding of the concept of peak VO_2_ in students aged 8–16 years. Our first research question asks if age is a significant factor that influences the level of understanding of peak VO_2_. As revealed by the total scores of the peak VO_2_ inventory, except for the youngest group of children (aged 8) who performed worse than the three older groups, no significant age effect was observed. Older participants did not always perform better than younger ones, a finding that did not conform to our expectation of an increasingly higher correct response rate with increasing age. This finding also suggests that the concept formation behind the understanding of peak VO_2_ cannot simply be explained by age-related cognitive development. Rather, peak VO_2_ is a concept that demands a multitude of reasoning skills to derive the factors affecting its volume, with a distinctive developmental pathway for each of these skills.

The participants' incorrect responses may be explained by their failure to form a possible causal link between antecedent (cause) and outcome (effect) for hypothetical situations. As mentioned, one critical skill for a proper understanding of peak VO_2_ may be prefactual thinking, a type of logical thought. Introducing the concept of peak VO_2_ through visualization, video simulation, multimedia learning materials, or real object models would be useful for students whose abstract reasoning skills are yet to fully developed. Given their underdeveloped knowledge of peak VO_2_, children and adolescents cannot be aware of its importance; even when this is explained to them, they cannot decide on what action may enhance peak VO_2_. Instead of general training in logical thinking, specific health-concept coaching is recommended in health education. One study demonstrated that people with a low aerobic capacity may have difficulty in sustaining more demanding activities (23), and another found that aerobic capacity contributed to cardiac workload and was associated with heart growth during childhood and adolescence (24).

Apart from age difference, we observed gender differences in choosing the answer out of four options, i.e., increase, decrease, unchanged, and uncertain. Female students were more inclined than male students to choose the answer option “uncertain” for the questions they responded incorrectly. Although we specified in the instruction that the answer option “uncertain” meant “the resulting peak VO_2_ is not certain,” the respondents might choose this answer option when they were indecisive about their decision. In future research, we suggest using another word (e.g., “insufficient information to determine”) to replace the word “uncertain.”

As our results demonstrate, students aged 8–16 years have not yet developed a satisfactory level of understanding of the concept of peak VO_2_. Given the importance of moderate-to-vigorous physical activity (MVPA), it is necessary to promote health literacy earlier than 8 years old. Therefore, further studies need to be done on younger children and examine their understanding of peak VO_2_. The inventory provided in this study would be useful for educators and psychologists to further develop age-appropriate assessment tool for testing younger children. Our study has also highlighted the importance of direct measure of students' understanding of health literacy-related concepts. As our study shows that age is not a strong predictor of the knowledge of peak VO_2_, we suggest educators not to assume children in the same class or grade have a similar level of understanding of peak VO_2_. Instead, we suggest to create a physical literacy profile for each student and keep a record of his/her current and improved understanding of all the essential concepts about physical literacy. In particular, we encourage the sport practitioners to demonstrate to children and adolescents the link between physical fitness and the volume of peak VO_2_. Given the abstractness of the concept, we suggest to test students using standard method (e.g. a progressive treadmill test) so that the experience of running and obtaining the result of peak VO_2_ can promote students' understanding of the concept. To further use the knowledge of peak VO_2_ to promote the awareness of maintaining an optimal level of peak VO_2_, regular measure and review of peak VO_2_ is recommended. Apart from running, sport practitioners are advised to refer to the Ecological Dynamics framework to design age-appropriate physical activities and environments that can support the improvement of peak VO_2_ (25). For instance, redesigning the playground in a school to encourage more moderate-to-vigorous physical activities during school time. As indicated in our findings, many children and adolescents mistakenly believed that many conditions can increase the peak VO_2_. Thus, it is necessary to clarify with these people the exact conditions in which peak VO_2_ increases and results an improvement of physical fitness. For the planning of health literacy curriculum, our findings of gender differences suggest the need to include suitable examples of physical activities that can arouse the interest in male and female students. Apart from school settings, it is also essential to promote the awareness and importance of the concept of peak VO_2_ in a wider community. As noted in previous studies (26), parent's physical literacy have strong influences on their children, especially the negative ones. For example, parents who have poor knowledge of physical literacy do not know how to overcome the barriers (e.g., lack of time and information) that hinder their participation in physical activities. When their children face a similar situation, these parents do not know how to overcome such challenges for their children. Therefore, we believe home-school cooperation is needed in order to promote regular participation of physical activities. While we assume that knowing will induce behaviors, future studies on the contribution of the understanding of peak VO_2_ to the actual participation of physical activity is warranted.

To conclude, children and adolescents ranging from 8 to 16 years old have suboptimal understanding of the antecedent of peak VO_2_. Given that age and gender have an effect (though not as consistent as we predicted) on the understanding of peak VO_2_, it is necessary to take chronological age and gender into account when assessing the mastery of this knowledge. It is critical to develop effective method for educating children and adolescents the concept of peak VO_2_ and the implications of poor peak VO_2_.

## Data Availability Statement

The datasets that support the findings of this study are available on request from the corresponding author. The datasets are not publicly available due to privacy or ethical restrictions.

## Ethics Statement

The studies involving human participants were reviewed and approved by Ethics committee of the Joint Chinese University of Hong Kong - New Territory East Cluster Clinical Research Ethics Committee. Informed and written consents were obtained from the student participants and their parents.

## Author Contributions

SW drafted the manuscript, performed the statistical analyses, and reviewed and revised the manuscript. CY coordinated and supervised data collection and entry and reviewed and revised the manuscript. AL obtained the funding for this project, supervised the data collection, and critically reviewed the manuscript. All the authors contributed to the article and approved the submitted version.

## Conflict of Interest

The authors declare that the research was conducted in the absence of any commercial or financial relationships that could be construed as a potential conflict of interest.
